# Medical Management of Interstitial Ectopic Pregnancy With Multidose Methotrexate

**DOI:** 10.7759/cureus.98111

**Published:** 2025-11-29

**Authors:** Soumya S Patil, Vidya M Jadhav, Falguni Mehta, Geeta Gore

**Affiliations:** 1 Obstetrics and Gynaecology, Bharati Vidyapeeth (Deemed to be University) Medical College and Hospital, Sangli, IND

**Keywords:** beta-hcg, interstitial ectopic pregnancy, methotrexate therapy for ectopic pregnancy, non-surgical management, transvaginal ultrasound scan

## Abstract

Interstitial ectopic pregnancy is a rare and potentially life-threatening type of ectopic pregnancy. Its location within the myometrial portion of the fallopian tube poses diagnostic challenges and increases the risk of hemorrhage upon rupture. We report the case of a 30-year-old primigravida who presented with lower abdominal pain and per vaginal spotting at approximately six weeks of gestation. Imaging confirmed a left interstitial ectopic pregnancy. She was hemodynamically stable and fulfilled the criteria for medical management. A multidose methotrexate regimen was administered, with serial monitoring of beta-hCG (human chorionic gonadotropin) and ultrasound findings. She received four doses of methotrexate. Her beta-hCG levels progressively declined, and imaging confirmed resolution. She remained stable throughout and resumed menstruation 26 days post-treatment. This case highlights the feasibility of medical management in interstitial ectopic pregnancy with strict monitoring protocols. Early diagnosis, appropriate patient selection, and fertility-preserving treatment strategies can lead to successful outcomes without surgical intervention.

## Introduction

Ectopic pregnancy refers to the implantation of a fertilized ovum outside the endometrial cavity. While tubal ectopics constitute the majority (approximately 95%), interstitial pregnancies represent a small subset of cases [1]. Interstitial pregnancy occurs in the proximal segment of the fallopian tube that is embedded within the uterine myometrium. Because this location allows for greater expansion of the gestational sac before rupture, diagnosis is often delayed, increasing the risk of severe hemorrhage and maternal mortality [2]. Early identification using transvaginal ultrasound and serial beta-hCG (human chorionic gonadotropin) measurements is critical [3]. Management options include surgical excision or conservative medical treatment, depending on the patient's stability, gestational age, and hCG levels [2,4]. In carefully selected cases, medical management with methotrexate offers an effective and fertility-preserving alternative [4,5].

## Case presentation

A 30-year-old woman, primigravida and married for four months, presented to our outpatient department with complaints of lower abdominal pain for five days and per vaginal spotting for two days. She had a spontaneous conception and was approximately five weeks and five days pregnant based on her last menstrual period. There was no significant medical, surgical, or allergy history. She was hemodynamically stable at presentation, with normal vital signs and systemic examination. Pelvic examination revealed an anteverted, normal-sized uterus with non-tender, free bilateral fornices.

**Figure 1:**
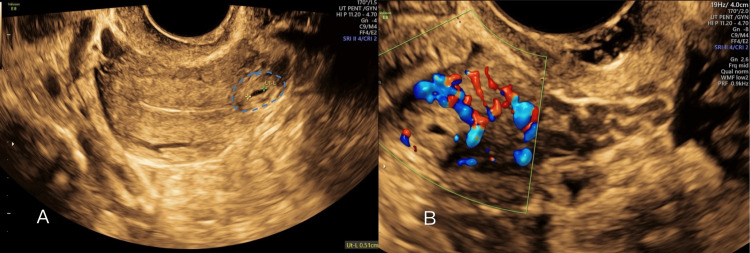
A) Transvaginal ultrasound showing 5 mm gestational sac in the left uterine cornu. B) Enhanced vascularity around the gestational sac

**Figure 2:**
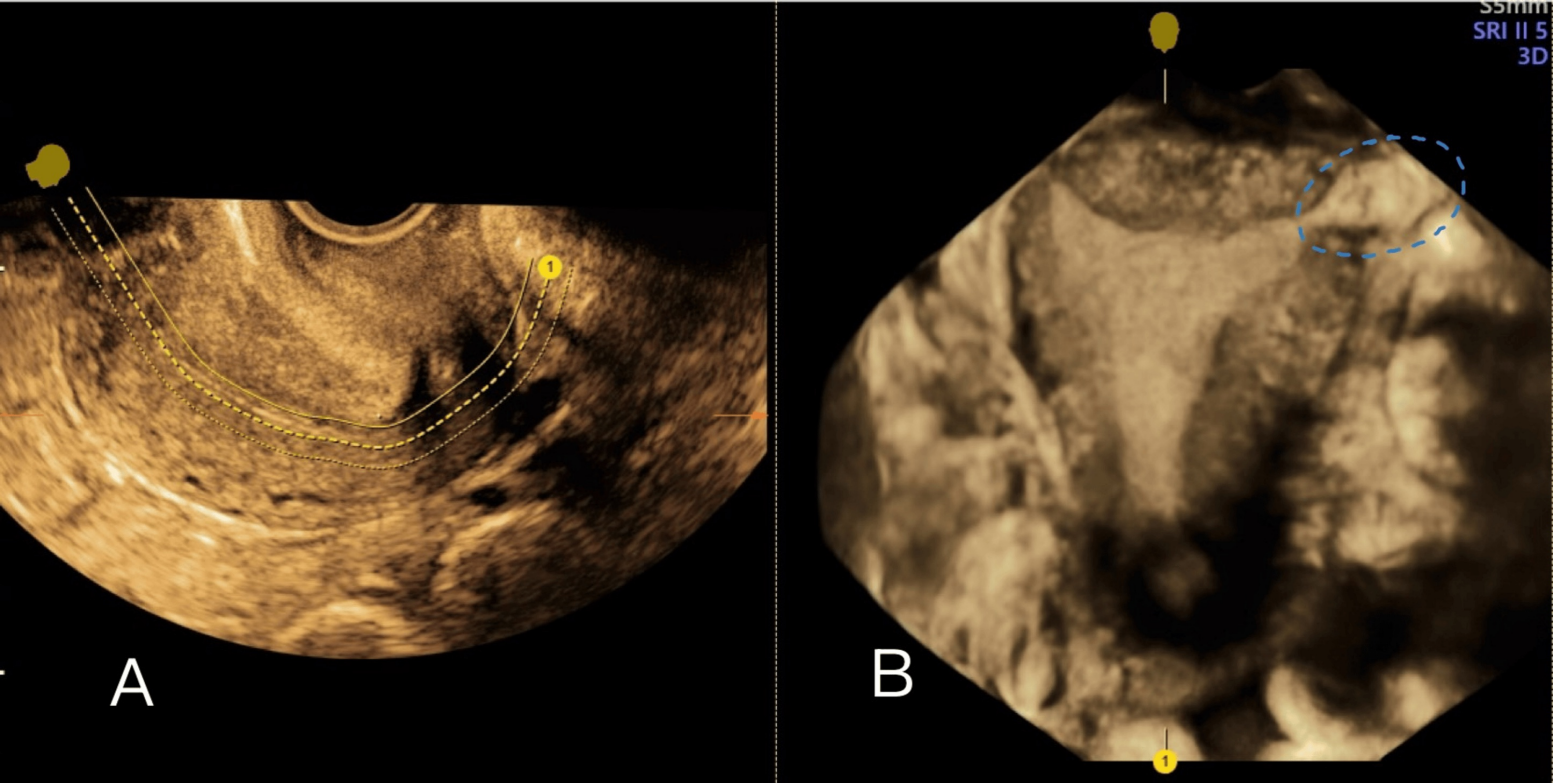
3D ultrasound surface rendering: A) empty uterine cavity and B) gestational sac in the left uterine cornu

A urine pregnancy test was positive. Initial transvaginal ultrasound showed a 5 mm gestational sac in the left uterine cornu without a fetal pole or yolk sac, suggestive of an early cornual ectopic pregnancy (Figure 1 and Figure 2).

**Figure 3:**
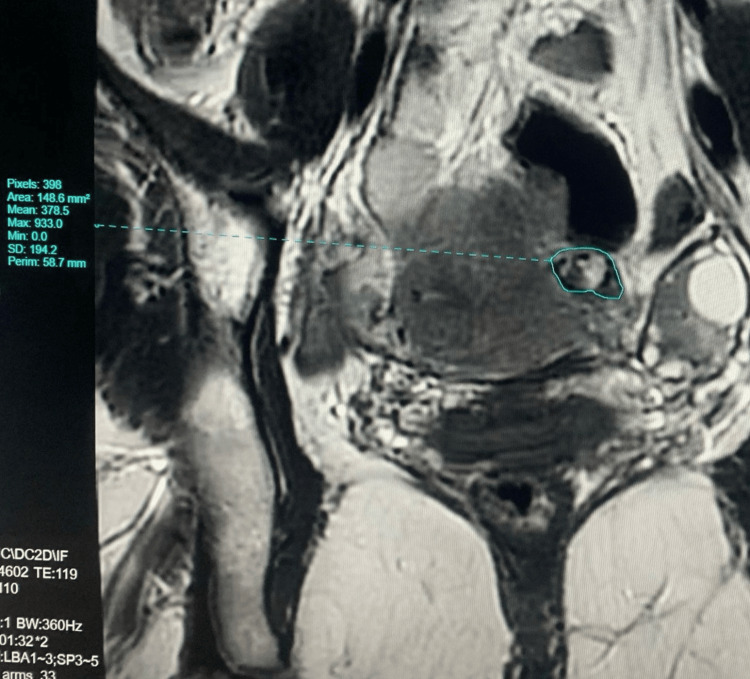
MRI image of left interstitial ectopic pregnancy: 17x12 mm-sized well-defined gestational sac is seen eccentrically located near the left uterine cornu

The patient delayed further evaluation for about two weeks. Ultrasound was repeated. Her baseline serum beta-hCG was 523.56 mIU/mL, and a repeat after 48 hours showed a mild increase to 566.35 mIU/mL (8.2% rise). MRI confirmed a well-defined gestational sac measuring 17×12 mm eccentrically located in the left uterine cornu, consistent with an interstitial ectopic pregnancy (Figure 3). She was admitted after the MRI evaluation. Laboratory tests, including complete blood count, liver function test, and renal function test, were all within normal limits (Table 1).

**Table 1:** Laboratory results Hb: hemoglobin; WBC: white blood cells; BUL: blood urea level; SGOT: serum glutamic oxaloacetic transaminase; SGPT: serum glutamic pyruvate transaminase.

Parameter	Reference range	Day 1	Day 4
Hb (mg/dL)	12-15	11.8	11
WBC (/mm^3^)	5000-10,000	5800	6400
Platelets (/mm^3^)	150,000-450,000	273,000	242,000
Blood sugar (mg/dL)	70-120	86	-
BUL (mg/dL)	12-45	15	-
Serum creatinine (mg/dL)	0.7-1.4	0.7	-
SGOT (U/L)	10-40	19	-
SGPT (U/L)	5-35	6	-
Total bilirubin (mg/dL)	0.1-1.2	0.8	-
Direct bilirubin (mg/dL)	0.1-0.4	0.3	-
Serum proteins (g/dL)	5.5-8.0	7.6	-
Serum albumin (g/dL)	3.5-5	4.5	-
Serum globulin (g/dL)	1.5-3	3.1	-

Medical management

**Figure 4:**
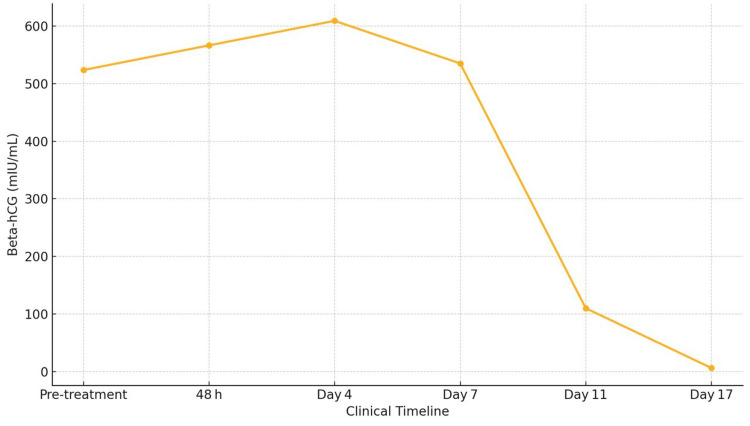
Beta-hCG trend during methotrexate therapy hCG: human chorionic gonadotropin.

On day 1, methotrexate was administered intramuscularly at a dose of 50 mg/m², with folinic acid at 0.1 mg/kg planned on alternate days (days 2, 4, 6, and 8). During treatment, the patient was monitored with serial serum beta-hCG levels on days 1, 4, 7, and thereafter weekly until resolution (Figure 4).

**Figure 5:**
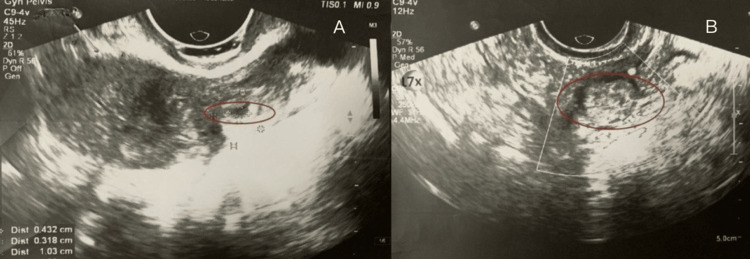
(A) Transvaginal ultrasound: Heterogeneous lesion on day 5 after the second dose of methotrexate. (B) Persistent blood flow noted within the lesion on color Doppler suggesting incomplete resolution

Complete blood count was repeated during therapy to assess for methotrexate toxicity. Clinical symptoms and vital signs were closely monitored throughout hospitalization and follow-up. On day 3, she received the second dose of methotrexate. On day 4, her beta-hCG was 609 mIU/mL, which did not show the expected decline, prompting continuation of treatment. On day 5, ultrasound showed a heterogeneous mass measuring 12×10 mm with cystic areas and a few vascular nidi, indicating incomplete resolution (Figure 5).

**Figure 6:**
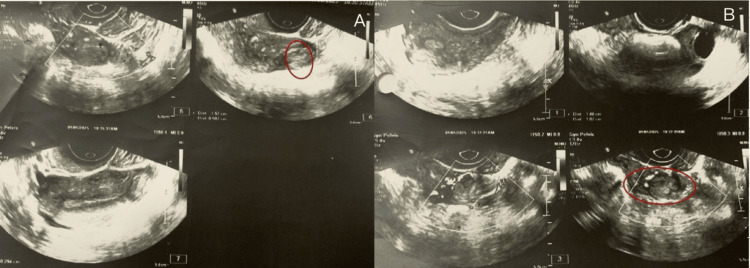
(A) Transvaginal ultrasound: Lesion regressed on day 7 after third dose of methotrexate. (B) No blood flow within the lesion on color Doppler with satisfactory response to methotrexate

She received the third methotrexate dose. On day 7, the lesion regressed to 15×9 mm with no cystic components or vascularity (Figure 6).

However, beta-hCG had only declined to 535 mIU/mL (12.2% drop), which was below the expected ≥15% fall, necessitating a fourth dose. The patient was discharged after receiving the fourth dose of methotrexate. By day 11, beta-hCG had decreased significantly to 108 mIU/mL. On day 18, beta-hCG further declined to 6 mIU/mL, suggesting near-complete resolution (Table 2).

**Table 2:** USG Findings, beta-hCG levels, interpretation, and treatment in interstitial ectopic pregnancy USG: ultrasonography or ultrasound; hCG: human chorionic gonadotropin.

Day	USG findings	Beta-hCG (mIU/mL; normal non-pregnant <5, viable pregnancy expected doubling every 48 hours)	Interpretation	Treatment given
Pretreatment finding	Small heterogeneous lesion in left cornu (11×11 mm)? Cornual/proximal tubal ectopic.	523.56 mIU/mL	Pretreatment finding	-
48 hours from baseline finding	No new USG, hCG only.	566.35 mIU/mL (after 48 hours) - 8.2% rise.	Mild rise in hCG. Smaller the increase in hCG level prior to administration of methotrexate, higher is the success.	-
Day 1	MRI finding - Gestational sac 17×12 mm in left cornu suggestive of left interstitial ectopic pregnancy.	-	Confirmed left interstitial ectopic pregnancy.	Methotrexate first dose given.
Day 3	-	-	-	Methotrexate second dose given.
Day 4	-	609 mIU/L (hCG repeated on day 4).	No expected fall in hCG.	Folinic acid second dose given.
Day 5	Heterogeneous mass 12×10 mm with cystic areas of size 4x3 mm with few vascular nidi within it.	-	Due to inadequate hCG drop, there was incomplete resolution.	Methotrexate third dose given.
Day 7	Lesion regressed to 15×9 mm, no cystic areas or vascularity.	535 mIU/L (hCG repeated on day 7)	Partial response to methotrexate (12.2% decrease. Expected to decrease by at least 15%).	Methotrexate fourth dose given.
Day 11	-	109.8 mIU/L	≥15% fall from 535 → satisfactory response.	-
Day 18	-	6 mIU/L	Continued appropriate decline post-treatment.	-

Throughout the treatment, the patient remained hemodynamically stable and compliant with follow-up, requiring no surgical intervention. She resumed spontaneous menstruation 26 days after completing methotrexate therapy. Follow-up included weekly beta-hCG monitoring until levels dropped below 15 mIU/mL, a repeat transvaginal ultrasound to confirm complete resolution, and a hysterosalpingogram after three months to assess uterine integrity. The patient was advised to avoid conception for at least three months post-treatment due to methotrexate effects and was counselled on a 2-5% recurrence risk of ectopic pregnancy. In her future pregnancy, early transvaginal ultrasonography will be essential to confirm intrauterine implantation.

## Discussion

The interstitial portion of the tube measures approximately 1-2 cm in length and is surrounded by myometrium, which complicates diagnosis and delays clinical recognition [1]. Because this location can accommodate a growing gestational sac, rupture tends to occur later than in other ectopic sites - typically between 8 and 16 weeks - often with catastrophic bleeding [1]. Interstitial pregnancy accounts for approximately 2% to 6.8% of all ectopic pregnancies [2]. Diagnosis is primarily achieved via transvaginal ultrasonography. Criteria include an empty uterine cavity, a gestational sac located laterally within the uterine wall, surrounded by a thin (<5 mm) rim of myometrium in all imaging planes, and the presence of the “interstitial line sign” [3]. MRI may serve as an adjunct in equivocal cases [4]. Medical management with methotrexate is appropriate in selected patients who are stable, with no evidence of rupture, and have low or plateauing beta-hCG levels and no fetal cardiac activity [5,6]. While single-dose regimens are often preferred, multidose regimens may be necessary in cases where beta-hCG fails to fall adequately [4]. Our patient met all eligibility criteria for methotrexate therapy. She required four doses of methotrexate due to an inadequate decline in beta-hCG by day 7. However, the pregnancy resolved without surgical intervention. Baseline liver function test and hematological profile were normal prior to methotrexate administration. Serial monitoring during therapy showed no evidence of hepatic impairment or hematological toxicity. This highlights the importance of close monitoring and patient compliance. Conservative management reduces the risk of surgical morbidity and preserves future fertility [7]. Early transvaginal ultrasonography is essential in subsequent pregnancies to exclude recurrence [8].

## Conclusions

Interstitial ectopic pregnancy poses a unique diagnostic and therapeutic challenge. With timely diagnosis and careful selection of patients, conservative management using multidose methotrexate can be safe and effective. Our case demonstrates successful resolution of an interstitial ectopic pregnancy with medical therapy alone, underscoring the role of strict monitoring, adherence to established guidelines, and fertility-preserving strategies in such cases.
